# Occurrence, partition and environmental risk assessment of per- and polyfluoroalkyl substances in water and sediment from the Baiyangdian Lake, China

**DOI:** 10.1038/s41598-020-61651-6

**Published:** 2020-03-13

**Authors:** Rui Guo, Xiaolei Liu, Jie Liu, Yan Liu, Xiaocui Qiao, Mengyu Ma, Binghui Zheng, Xingru Zhao

**Affiliations:** National Engineering Laboratory for Lake Pollution Control and Ecological Restoration, State Environmental Protection Key Laboratory of Drinking Water Source Protection, Chinese Research Academy of Environmental Sciences, Beijing, 100012 China

**Keywords:** Environmental monitoring, Environmental impact

## Abstract

This work examined the contamination of poly- and perfluorinated compounds (PFASs) in the water and sediment of the Baiyangdian Lake. The total concentration of PFASs in the surface water varied from 140.5 to 1828.5 ng/L, and the highest concentration of PFASs were observed near the entrance of Fuhe river. The topmost contaminant was sodium perfluorohexanesulfonate (PFHxS) and perfluorooctanoic acid (PFOA) in the north and south of the Baiyangdian Lake respectively, which indicated different contamination sources. The total concentration of PFASs in the sediment varied from 0.48 to 30 ng/g, and the distribution of PFASs in the sediment was similar with that in the surface water. The concentrations of polyfluoroalkyl phosphoric diesters (diPAPs) were three to four orders of magnitude lower than those of perfluorocarboxylates (PFCAs) and PFSAs. Although the pore water and the surface water had similar ΣPFASs, the concentration of perfluorodecanoic acid (PFDA) in pore water was 1.4 to 4.4 times higher than that in surface water, and the concentration of perfluoropentanoic acid (PFPeA) in pore water was 20–70% that in surface water. The results of ecological risk assessment showed that the PFASs were currently of no immediate risk to the aquatic life.

## Introduction

Perfluoroalkyl and polyfluoroalkyl substances (PFASs) are anthropogenic chemicals that are both oleophobic and hydrophobic. Their excellent surface activity and repellence to water, oil, and stain made them suitable for extensive applications in fire-fighting foams, defoaming additives, adhesives, cosmetics, cleaners, coatings, paints, electronics, as well as the production of fluoropolymers. Perfluoroalkyl carboxylates (PFCAs) and perfluoroalkanesulfonates (PFSAs) have been produced and utilized for more than 50 years and are readily detected globally in water, soil, sediment, wildlife, and human blood^1–7^. In May 2009, the Stockholm Convention listed perfluorooctane sulfonic acid (PFOS), its salt and perfluorooctane sulfonyl fluoride as persistent organic pollutants (POPs)^8^.Unfortunately, short chain PFCAs and PFSAs are nowadays detected in rising concentration because they are used as replacements of PFOS and perfluorooctanoic acid (PFOA), although they are also persistent in the environment or degrade into persistent molecules^9^.To make things worse, short chain PFASs are harder to remove from surface water, and data are lacking on their health effect^10^.

In contrast, emerging per- and polyfluoroalkyl substances are much less well understood in terms of their environmental occurrence and fate. These compounds include perfluoroalkyl phosphonic acid (PFPAs), perfluoroalkyl phosphinic acids (PFPiAs), polyfluoroalkyl phosphoric acid monoesters (monoPAPs), polyfluoroalkyl phosphoric diesters (diPAPs), *etc*.Among them, PFPAs and PFPiAs have in fact been produced since the 1970s and used in pesticides as wetting agents, leveling agents and defoaming agents^11,12^, and were among the perfluoroalkyl acids of high production volume (4500–227000 kg/yr) in 1998 and 2002^13^. It was proven that PFPiAs could biodegrade into the corresponding PFPAs of the same chain length in rainbow trout^14^. In addition, it was suggested that PFPA and PFPiAs could be potential precursors of PFCAs. On the other hand, diPAPs have been produced since the 1970s and used in paper, packaging materials, cosmetics, and cleaning agents^15^. These compounds have been detected in effluent wastewater, human serum, indoor dust, fish mussels, and raptors^16–22^. Unfortunately, compared to PFCAs and PFSAs, little is known about the environmental occurrence and fate of PFPAs and diPAPs, particularly in North China.

Besides insufficient data about PFPAs and diPAPs in lakes of China, Sediment-porewater partition of PFASs, which is an important process that controls the transport, fate, and ecotoxicological risk of the micro-levels of contaminants in aquatic environments are also inadequate. Studies have investigated sediment-porewater partition of polycycle aromatic hydrocarbons (PAHs)^23^, polychlorinated biphenyl (PCB) congeners^24^, Mercury and methylmercury^25^, etc., however, reports of PFASs in porewaters and their sediment-porewater partition were still unknown. In order to better understand the mobility and bioavailability of PFASs contained in the sediments, the concentration of these compounds in the sediment porewaters must be determined.

The Baiyangdian Lake, known as “Pearl of North China”, is the largest wetland in North China. It plays an important role in flood control, water storage, climate regulation, and improvement of ecological environment. With the rapid economic development and rising population in recent years, the water quality of the Baiyangdian Lake deteriorated gradually. The Baiyangdian Lake and its surrounding areas have 39 villages with about 243,000 inhabitants, with a large amount of daily raw domestic sewage discharged into the Baiyangdian Lake. Besides, a large number of domestic sewage and industrial wastewater from the Baoding City in the upper reaches enters the Baiyangdian Lake directly via the Fuhe River. As a result of such intensive anthropogenic and industrial activities, heavy metal, organochlorine pesticides (OCPs) and polybrominated diphenyl ethers (PBDEs) have been detected in this area^26,27^. Though there were several publications on PFSAs levels in Baiyangdian Lake environmental samples^28–30^, few reported the distribution of PFSAs between sediment and pore waters. Further more, little is known about the pollution of per- and polyfluoroalkyl substance in the past ten years, especially PFPAs, PFPiAs, and diPAPs. The current study analyzed PFASs contamination level in collected sediment, pore water, surface water samples from Baiyangdian Lake. And the partitioning of PFSAs between sediment and pore water were explored to determine the potential factors affecting their environmental behavior and fate. To our best knowledge, this is the first report of PFASs partitioning between sediment and pore water and contamination levels of PFPAs, PFPiAs and diPAPs in Baiyangdian Lake.

## Materials and Methods

### Chemicals and reagents

All reference and mass-labeled compounds were purchased from Wellington Laboratories (Ontario, Canada).The PFCAs standards included perfluorobutanoic acid (PFBA), perfluoropentanoic acid (PFPeA), perfluorohexanoic acid (PFHxA), perfluoroheptanoic acid (PFHpA), perfluorooctanoic acid (PFOA), perfluorononanoic acid (PFNA), perfluorodecanoic acid (PFDA), perfluoroundecanoic acid (PFUnA), perfluorododecanoic acid (PFDoA), perfluorotridecanoic acid (PFTrA), and perfluorotetradecanoic acid (PFTeA),.The PFSAs standards contained potassium perfluorobutanesulfonate (PFBS), sodium perfluorohexanesulfonate (PFHxS), sodium perfluorooctanesulfonate (PFOS), and sodium perfluorodecanesulfonate (PFDS). The PFPAs and PFPIAs standards included perfluorohexylphosphonic acid (C6-PFPA) perfluorooctylphosphonic acid (C8-PFPA), perfluorodecylphosphonic acid (C10-PFPA), sodium bis(perfluorohexyl)phosphinate (C6/C6-PFPIA), sodium perfluorohexylperfluorooctylphosphinate (C6/C8-PFPIA), and sodium bis(perfluorooctyl)phosphinate (C8/C8-PFPIA). The DiPAPs standards contained sodium bis(1H, 1H, 2H, 2H-perfluorooctyl)phosphate (6:2 diPAP) and sodium bis(1H, 1H, 2H, 2H-perfluodecyl)phosphate (8:2diPAP). The mass-labeled internal standard (IS) included [^13^C_4_]-PFBA, [^13^C_2_]-PFHxA, [^13^C_4_]-PFOA, [^13^C_5_]-PFNA, [^13^C_2_]-PFDA, [^13^C_2_]-PFUnDA, [^13^C_2_]-PFDoDA, [^18^O_2_]-PFHxS, [^13^C_4_]-PFOS, [^13^C_4_]-6:2 diPAP, and [^13^C_4_]-8:2diPAP.

LC-MS grade methanol, acetonitrile, and methyl-*tert*-butyl ether (MTBE) were purchased from Fisher Scientific (Fair Lawn, NJ, USA). LC grade ammonium acetate waspurchased from Fisher Scientific (Fair Lawn, NJ, USA). Analytical grade sodium hydroxide (NaOH) was purchased from Sinopharm Chemical Reagent Beijing, Co., Ltd. Tetrabutyl ammonium hydrogen sulphate (TBAS) was purchased from J.T. Baker (Phillipsburg, NJ, USA). Oasis^®^ weak anion exchange solid phase extraction cartridges (WAX; 6 cc, 150 mg) were purchased from Waters (Milford, MA). Milli-Q water was used in all analytical experiments.

### Sample collection

Surface water and sediment samples were collected at Baiyangdian Lake in March 2016. Surface water samples were collected with a stainless steel bucket and stored in 1 L polypropylene (PP) containers with a narrow mouth and a screw cap. A total of 15 surface water samples were collected. The corresponding sediment samples were collected with a bottom grab and stored in stainless steel containers.

**Figure 1:**
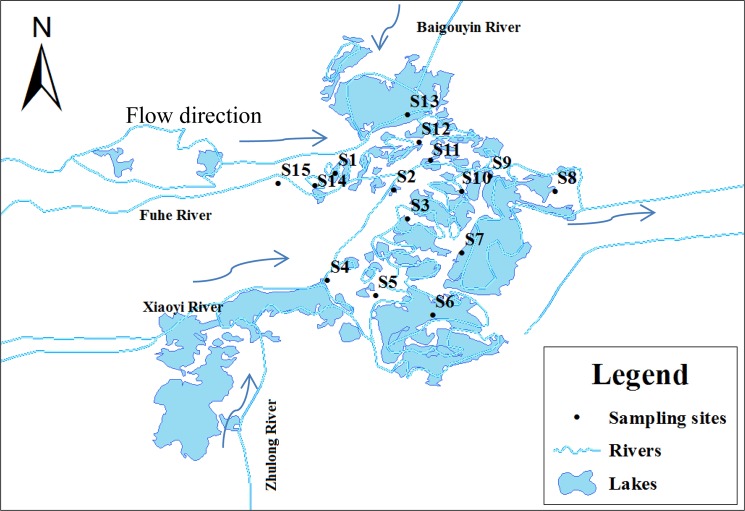
Sampling sites at the Baiyangdian Lake area.

Sample duplicates and field blanks were collected and analyzed along with laboratory and procedural blanks. The stainless steel bucket, stainless steel containers, and PP bottles were cleaned before use by rinsing sequentially with methanol, distilled water, and then water from the sampling site. All samples were kept in an ice bath during shipping, and all water samples were extracted immediately upon arrival at the laboratory. Figure 1 illustrates the sampling sites.

### Sample preparation

#### Water samples

Both surface water samples and pore water samples were prepared. Pore water samples were collected by centrifuging the sediment samples from S3, S4, S6–S11, S13, and S15 at 10000 rpm for 10 min. Water samples were prepared according to previously published methods^31^. Water samples were filtered with Whatman GF/F glass microfiber (0.7 μm pore size, 4.7 cm diameter) and then extracted with Oasis WAX solid phase cartridges (150 mg, 6cc, Waters, MA, USA). Before solid phase extraction, each mass-labeled PFSA (2 ng) was spiked into the water sample as internal standard. The WAX cartridge was conditioned with 1% NH_4_OH in methanol (6 mL), followed by methanol (6 mL) and Milli-Q Water (6 mL). Water samples were passed through the conditioned cartridge at 5 mL/min, and the loaded cartridge waswashed firstly with ammonium acetate buffer (pH = 4, 25 mmol/L, 6 mL) and then with MeOH (2 mL). The target compounds were eluted firstly with 1% NH_4_OH in methanol (6 mL) and then with 1% NH_4_OH in acetonitrile (6 mL). The eluate was then blown to dryness with a gentle stream nitrogen gas and dispersed in methanol (1 mL).

#### Sediment samples

Sediment samples were freeze-dried, homogenized with mortar and pestle, and extracted according to the reported ion-pairing method^32^. Individual internal standards (2 ng each) were spiked into sediment samples before extraction.

The extracts were separated into three fractions before instrumental analysis to enhance the sensitivity for target compounds. The first fraction, with which PFCAs and PFSAs were analyzed, was blended with Milli-Q water to become a solution of 1:1 v/v methanol/water. The second fraction, with which PFPAs were analyzed, was adjusted similarly to become a 1:1 mixture of methanol and 25 mmol/L TBAS. No adjustment was made to the third fraction, with which diPAPs and PFPIAs were analyzed.

### Instrumental analysis

Target compounds were separated using Ultra-Pressure Liquid Chromatography (UPLC) coupled with a Micromass Xevo-TQD mass spectrometer (UPLC-Xevo-TQD, Waters, USA) operated in the negative electrospray ionization mode. The injection volume was 10 µL. The analytes were separate on a Waters Acquity BEH C18 column (50 mm × 2.1 mm i.d., 1.7 μm) using aqueous ammonium acetate and methanol as the mobile phase with agradient elution program similar to those reported elsewhere. Multiple reactions monitoring of target compounds and optimized mass spectrum parameters were also similar to those reported elsewhere^32^.

Gradient elution was used to separate different compounds by liquid chromatography.For the analysis of PFCAs and PFSAs, the mobile phase consisted of (A) 10 mmol/L ammonium acetate in HPLCgrade water and (B) 10 mmol/L ammonium acetate in 8:2 (v/v) methanol/acetonitrile. To analyze PFPiAs, diPAPs, and PFPAs, the mobile phase consisted of 0.1% NH_4_OH in HPLC grade water (A) and pure methanol (B). The flow rate was 300 μL/min and the injection volume was 10 μL. Multiple reaction monitoring (MRM) of target compounds and optimized mass spectrum parameters followed a reported set of conditions^33^.

The total organic carbon content (TOC) of water and sediment samples was analyzed using a multi N/C 2100 S system (AnalytikJena, Germany) with a procedure similar to those described elsewhere^32^.

### Quality assurance and quality control

Quality assurance and control measures included field blank, travel blank, procedural blank, calibration curve, spike recoveries (both blank and matrix), and limit of quantification (LOQ). Field blanks were prepared by filling precleaned 1 L collection bottles with laboratory Milli-Q water that was previously determined to be free of PFSAs. Procedural blanks were analyzed with every batch of samples. Procedural and travel blanks were below the corresponding LOQs. Analyte recoveries were checked to determine the accuracy of the methods. Matrix spike recovery tests were performed for both water and sediment. To reduce instrumental background contamination from HPLC or solvents, an isolate trap column was connected between the solvent mixing cell and the six-way valve. Teflon-coated lab ware and glassware were avoided during all steps of sampling, pretreatment, and analysis to minimize contamination. The limits of quantification (LOQs) were defined as the smallest mass of injected compound that could afford a reproducible measurement of peak area within ±20% of the duplicate injection. The LOQ and recoveries for each compound were shown in Table 1. The PFASs concentrations were quantified using external calibration curves consisting of a concentration series of 0, 10, 20, 50, 100, 200, 1000, and 2000 ng/L, and the deviation of every point from the regression line was less than 20% from its theoretical value.

**Table 1:** Limits of quantification and recoveries of target compounds.

Compounds	LOQ (ng/L)	Recoveries (%)	
		water	sediment
PFBA	50	98.1 ± 10.8	68.8 ± 9.6
PFPeA	50	81.8 ± 8.9	76.3 ± 9.1
PFHxA	50	103.1 ± 9.11	75.5 ± 10.5
PFHpA	20	97.2 ± 12.0	74.2 ± 7.6
PFOA	50	96.8 ± 3.3	76.4 ± 10.4
PFNA	100	103.5 ± 4.6	75.5 ± 8.4
PFDA	20	75.6 ± 2.9	75.8 ± 7.4
PFUnA	50	80.1 ± 2.5	77.3 ± 5.4
PFDoA	20	107.0 ± 7.1	87.5 ± 10.2
PFTrA	50	75.3 ± 7.8	102.3 ± 3.5
PFTeA	50	58.6 ± 11.5	94.6 ± 6.4
PFBS	100	67.5 ± 6.8	69.4 ± 7.6
PFHxS	100	105.4 ± 10.1	89.6 ± 8.5
PFOS	50	105.8 ± 5.5	85.4 ± 9.8
PFDS	100	60.8 ± 10.5	97.6 ± 11.5
C6-PFPA	100	103.9 ± 9.1	80.2 ± 3.6
C8-PFPA	100	104.6 ± 8.2	75.6 ± 2.6
C10-PFPA	100	89.7 ± 10.8	74.5 ± 3.2
C6/C6 PFPIA	20	66.5 ± 12.5	86.6 ± 2.5
C6/C8 PFPIA	20	54.2 ± 12.2	90.5 ± 2.4
C8/C8 PFPIA	20	89.2 ± 13.3	85.7 ± 2.6
6:2 diPAP	20	108.3 ± 8.8	78.5 ± 5.7
8:2 diPAP	20	115.2 ± 6.9	82.4 ± 7.6

### Assessment method for environmental risk of PFASs

The environmental risk of the PFASs was assessed based on their risk quotient (RQ), currently the most commonly used measure to characterize environmental risk, calculated by dividing the measured concentration of the target substance (MEC) with the predicted no-effect concentration (PNEC):

$${\rm{RQ}}=\text{MEC}/\text{PNEC}$$

Risk level was judged as follows: RQ < 0.01, very low risk; 0.01<RQ < 0.1, low risk; 0.1<RQ < 1, intermediate risk; RQ > 1, high risk.

Because toxicological data of PFASs in sediment are lacking, the sediment PNEC was calculated based on equilibrium distribution. Thus, according to the technical guidance document of the European Union for the risk assessment of chemical substances (TGD)^34^.

## Results and Discussions

### PFASs in surface water

Among the 15 analyzed PFCAs and PFSAs, ten were detected in the water samples. The concentrations of PFUnA, PFDoA, PFTrA, PFTeA and PFDS were lower than the LOQs.

**Figure 2:**
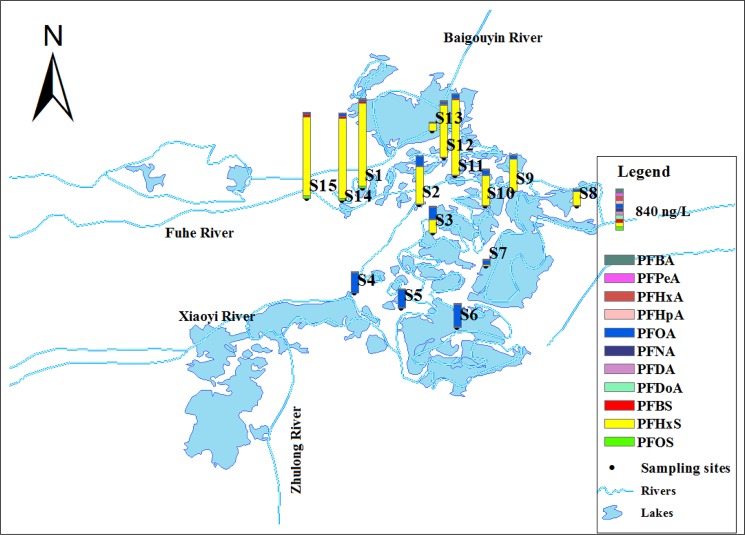
Distribution of PFCAs and PFSAs in Baiyangdian Lake surface water.

Figure 2 shows the distribution of PFASs in the surface water samples from the Baiyangdian Lake. The total concentration of the PFCAs and PFSAs varied from 140.5 to 1828.5 ng/L. Among the tested compounds, C6–C8, C10 PFCAs and C6, C8 PFSAs were detected in all water samples. The detection rate of the PFASs with shorter chains,*i.e*., PFBA, PFPeA, PFNA, and PFBS,reached 66.7%, 80%, 80%, and 93.3%, respectively. The contaminant level of the target compounds decreased in the following order: PFHxS (range: 2.07–1688 ng/L, mean: 684 ng/L) > PFOA (range: 13.6–441 ng/L, mean:147 ng/L) > PFBS (range: nd–51.2 ng/L, mean: 16.9 ng/L) > PFOS (range: 0.58–51.2 ng/L, mean: 15.2 ng/L) > PFPeA (range: nd–12.7 ng/L, mean: 5.97 ng/L) > PFHxA (range: 2.36–6.12 ng/L, mean: 4.84 ng/L) > PFHpA (range: 1.16–9.5 ng/L, mean: 3.16 ng/L) > PFBA (range: nd–5.25 ng/L, mean: 2.22 ng/L) > PFNA (range: nd–1.02 ng/L, mean: 0.61 ng/L) > PFDA (range: 0.254–0.762 ng/L, mean: 0.39 ng/L). There must be different contamination sources since PFOA was dominant at S4–S7 however, PFHxS was dominant at other sites. The sites S4–S6 are near the entrance of the Xiaoyi River and the Zhulong River. As one of nine rivers entering the Baiyangdian Lake, the Zhulong River carries abundant wastewater from textile and fur plants and had a high PFOA concentration up to 8397.23 ng/L^29^. The highest concentrations of total PFASs were detected at S14, S15, and S1, which are near the entrance of the Fuhe River. The Fuhe River passes through the Baoding city and carries untreated urban sewage as it flows into the Baiyangdian Lake, and it is thus a principal source of pollution^35^. A photographic film production plant from one of the largest Chinese manufacturers is by the Fuhe River, and previous studies showed that the Fuhe River contained abundant PFHxS (>1000 ng/L). The pollutants in the samples from sites other than S4–S7 showed a similar distribution pattern (Fig. 2), which hinted on a common contamination source. The study of the Baiyangdian Lake in 2016 showed that PFOA (up to 8397 ng·L − 1) and PFHxS (up to 1478 ng·L − 1) were the predominant PFASs detected in the surface water^29^, which indicated the common contamination source of S1-S3, S8-S15. The PFSAs concentrations of the surface water collected from the Baiyangdian Lake in October 2010 ranged in 14.8–95.6 ng/L, and the lowest concentration detected in the current study was even higher than the highest concentration previously reported^30^. The comparison indicated that contamination of PFSAs in the Baiyangdian Lake deteriorated since 2008.

**Figure 3:**
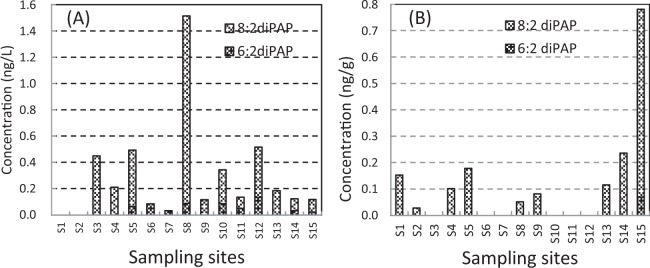
Distribution of diPAPs in the Baiyangdian Lake. (**A**) surface water, (**B**) sediment.

All surface water samples were free of PFPAs and PFPiAs, and 6:2 diPAP and 8:2 diPAP were detected in 60% and 73% of the surface water samples, respectively. The concentration ranged in nd–0.134 ng/L and nd–1.43 ng/L for 6:2 diPAP and 8:2 diPAP, respectively,three or four orders of magnitude lower than the concentrations of PFCAs and PFSAs (Fig. 3). The concentration of 8:2 diPAP was clearly much higher than that of 6:2 diPAP. The diPAPs must have come from a contamination source different from that of PFCAs and PFSAs because of their distribution characteristics and the higher concentration of 8:2 diPAP. Since PAPs are primarily used in paper products for food packaging, the diPAPs probably came from domestic sewage and household garbage^36^. Since the degradation of diPAPs to PFCAs can occur in wastewater treatment plants, diPAPs must be both a precursor of PFCAs and a potential fluorinated contaminant of their own^37,38^. To the best of our knowledge, this work is the first report that determined diPAPs in the Baiyangdian Lake.

The regression analyses of the contaminants in the surface water of the research area indicated that significant correlations (*p* < 0.05) were found between various compounds. Especially, the correlations between PFOS and PFHxS, PFOS and PFBS, as well as PFHxS and PFBS were all more than 0.93, *i.e*., these three compounds might share similar sources and transport routes.

### PFSAs in pore water

**Figure 4:**
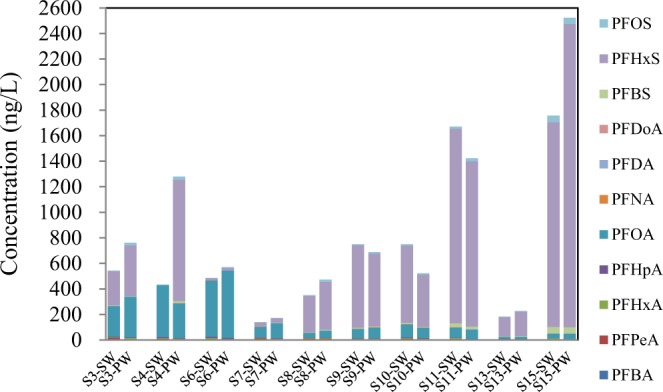
Comparison of total concentration of PFCAs and PFSAs in the surface water and the pore water of the Baiyangdian Lake, SW stands for surface water, PW stands for pore water.

The distribution characteristics of PFCAs and PFSAs in pore water were shown in Fig. 4. Since the content of dissolved organic matter (DOM) in pore water was usually more than one order of magnitude than that in surface water, it was excepted the total concentration of PFASs in pore water would higher than those in surface water. However, the results displayed in Fig. 4 didn’t support the expectation. As shown in Fig. 4, total concentration of PFASs in pore water of S3, S4, S6, S7, S8, S13 and S15 were higher than those in correspond surface water, while in sampling sites S9, S10 and S11, the results was in the opposite.

water and the pore water of the Baiyangdian Lake, SW stands for surface water, PW stands for pore water

**Figure 5:**
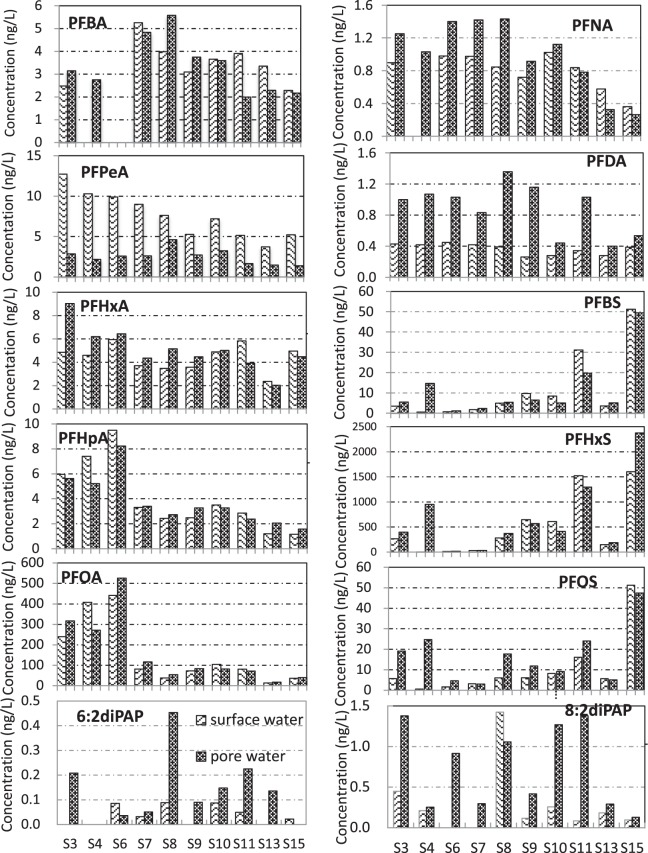
Distribution of PFCAs and PFSAs between surface water (sw) and pore water (pw) in the samples from the Baiyangdian Lake.

Figure 5 presents the distribution of each PFSA congener in the surface water and the pore water. The concentration of PFPeA was much higher in surface water than in pore water, whereas PFDA was more enriched in pore water. Interestingly, although PFBA has a shorter carbon chain and presumably higher solubility in water, it was not enriched in surface water. The distribution of PFHxA, PFHpA, PFOA, PFBS, PFHxS, and PFOS showed a varied preference between surface water and pore water. The 6:2 diPAP and 8:2 diPAP were enriched in pore water in most cases.

### PFASs in sediment samples

**Figure 6:**
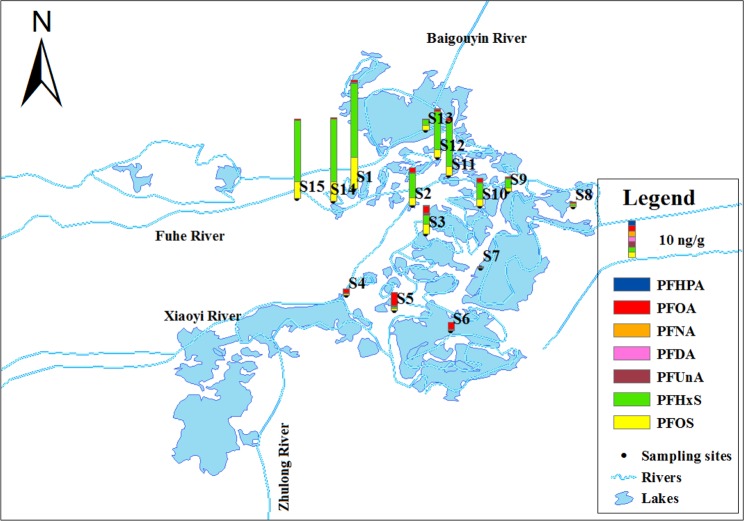
PFSAs contamination in sediment samples from the Baiyangdian Lake.

Among the 23 target compounds, four PFCAs (PFHpA, PFOA, PFNA and PFDA), two PFSAs (PFHxS and PFOS), and both diPAPs were detected in sediment samples. Figure 6 shows the concentrations of these compounds in the sediments at 15 sampling sites of the Baiyangdian Lake. The detection rate of PFCAs and PFSAs decreased in the order of PFOS (100%, 0.11–8.59 ng/g) = PFOA (100%, 0.16–3.67 ng/g)>PFHxS (93%, nd–20.5 ng/g)> PFNA (66.7%, nd–0.11 ng/g)> PFDA (60%, nd–0.19 ng/g)>PFHpA (46.7%, nd–0.23 ng/g). The distribution profile of the PFCAs and PFSAs in sediment was similar to that of water. Specifically, PFOA was dominant at S4–S7, and PFHxS was dominant in all other sediment samples. Note that PFBA, PFPeA, PFHxA, PFBuS were detected in most surface water samples with a mean concentration from 2.22 to 16.9 ng/L but not detected in sediment samples, possibly because of their low affinity to sediments.

Comparison with literature showed that the observed PFASs concentration at the Baiyangdian Lake was much higher than the ΣPFASs of the Songhua River (0.143–1.41 ng/g), the Peal River estuary (nd–2.41 ng/g), the Nansi Lake (0.47–1.81 ng/g), and the Daliao River (0.13–0.49 ng/g)^39–42^. In whole worldwide, the ΣPFASs in sediment varied greatly. The ΣPFASs level in sediment of target area was similar with those in Lake Superior (nd–10.5 ng/g), Lake Huron (nd–26.0 ng/g) and Lake Michigan (0.2–10.1 ng/g) collected in 2011 and 2012 in North America^43^ and urban catchment of Singapore (1.6 to 15 ng/g d.w)^44^. Besides, the sediment concentrations of PFOS and PFOA were at an intermediate level compared with the situations of other parts of the world. However, the observed PFHxS concentration was at a relatively high level.

### Sediment-water diffusion

The partition of organic pollutants between water and sediment affects their environmental behavior and fate, and the partition is governed by their physical and chemical properties as well as sediment characteristics such as organic carbon content, pH, ionic strength, salinity, *etc*.^45^. In current study, we calculated the distribution coefficient (*K*_*d*_) of PFOA, PFOS and PFHxS between sediment and surface water as well as between sediment and pore water. We only calculated *K*_*d*_ for PFOA, PFOS and PFHxS because their detection rate was > 90%. Significant correlations (p < 0.01) were found between sediment TOC and the sediment–pore water *K*_*d*_ of PFHxS, PFOA, and PFOS (Tables 2–4).

**Table 2:** Correlations of PFOA.

		K_d_	TOC
K_d_	Pearson Correlation	1	0.802(**)
	Sig. (1-tailed)		0.003
	N	10	10
TOC	Pearson Correlation	0.802(**)	1
	Sig. (1-tailed)	0.003	
	N	10	10

**Significant correlation at p < 0.01 (1-tailed).

**Table 3:** Correlations of PFOS.

		K_d_	TOC
K_d_	Pearson Correlation	1	0.862(**)
	Sig. (1-tailed)		0.001
	N	10	10
TOC	Pearson Correlation	0.862(**)	1
	Sig. (1-tailed)	0.001	
	N	10	10

**Significant correlation at p < 0.01 (1-tailed).

**Table 4:** Correlations of PFHxS.

		K_d_	TOC
K_d_	Pearson Correlation	1	0.862(**)
	Sig. (1-tailed)		0.001
	N	10	10
TOC	Pearson Correlation	0.862(**)	1
	Sig. (1-tailed)	0.001	
	N	10	10

**Significant correlation at p < 0.01 (1-tailed).

There was no significant correlation (p > 0.01) between sediment TOC and the sediment–surface water *K*_*d*_ of the tested compounds. Hence, after equilibrium was established for the distribution of the compoundsbetween pore water and sediment, the sediment TOC would become the dominant factor affecting the distributionof the pollutant between pore water and sediment.

The partitioning of PFASs is commonly evaluated by *K*_*oc*_ as follows:

$${{\rm{K}}}_{{\rm{oc}}}=({{\rm{C}}}_{{\rm{s}}}{/C}_{{\rm{w}}}){/f}_{{\rm{oc}}}$$

where C_s_ is the PFASs concentration of the entire sediment based on dry weight (ng/g); C_w_ is the PFASs concentration of surface or pore water at equilibrium (ng/mL); and *f*_*oc*_ is the organic carbon fraction of the sediment (%). The log*K*_*oc*_ values of the PFASs in pore water were: PFOA, 2.05–2.56 (mean 2.21), PFHxS, 1.82–2.32 (mean 2.09), PFOS, 2.62–3.53 (mean 3.24). The log*K*_*oc*_ values of the PFASs in surface water were: PFOA, 1.89–2.71 (mean 2.24), PFHxS, 1.94–3.28 (mean 2.38), PFOS, 3.01–4.25 (mean 3.59). It could be seen from the results of PFHxS and PFOS that each –CF_2_ group contributed 0.58–0.60 log units to the log*K*_*oc*_ value, which was consistent with previous reports^32,46^.

### Environmental risk of PFASs: an assessment

The RQ of PFASs were calculated to evaluate their environmental risk.

The PNEC_water_ of PFOA, PFOS, PFNA, PFHxA, and PFDA were 100, 25, 100, 97, and 11 µg/L, respectively^47^. The PNEC_sediment_ of PFOA and PFOS were 2060 and 67 µg/kg, respectively. The RQ_water_ of PFOA, PFOS, PFNA, PFHxA, and PFDA were all < 0.01, which indicated very low environmental risk. The RQ_sediment_ of PFOA was <0.01 but the RQ_sediment_ of PFOS ranged in 0.002–0.13. Therefore, the environmental risk level of PFOS varied from very low to intermediate.

Since current toxicity data of PFHxS and PFBS indicate that they are less toxic than PFOS and PFOA, it was expected that they should have higher PNEC than PFOS^48–52^. The measured concentration of PFHxS and PFBuS in water and sediment were even lower than the PNEC of PFOS. Hence, PFHxS and PFBS in the Baiyangdian Lake did not have immediate environmental impact on the aquatic life.

The results showed that the PFASs in the Baiyangdian Lake would not generate immediate environmental impacts on the aquatic life. Nevertheless, uncertainty might arise in assessing the ecological risk of PFASs and their impact on aquatic organisms due to the scarcity of toxicity information and toxicity data of pollutants mixture, and also because PNEC values derived from different methods may vary. As a result, comprehensive toxicological information, systematic environmental exposure data, along with realistic risk assessment method are all needed to address the adverse effects of PFASs on aquatic systems.

## Conclusions

This study investigated the contamination of poly- and perfluorinated compounds (PFASs) in the water and sediment of the Baiyandian Lake with a careful analysis of their distribution, partitioning, as well as environmental hazard. The results showed that the concentration of perfluoropentanoic acid (PFPeA) was much higher in surface water than in pore water, and no significant difference was otherwise found in the total concentration of PFASs between surface water and pore water. In surface water, the total concentration of perfluoroalkyl carboxylates (PFCAs) and perfluoroalkanesulfonates (PFSAs) varied from 140.5 to 1828.5 ng/L.The dominant congener was sodium perfluorohexanesulfonate (PFHxS) and perfluorooctanoic acid (PFOA) in the north and south of the Baiyangdian Lake, respectively. The highest PFSAs concentration was detected near the Fuhe River, which appeared to be an important source of contamination. All sediment samples contained perfluorooctane sulfonic acid(PFOS) and perfluorooctanoic acid (PFOA). It was found that the PFASs did not pose immediate environmental risk at the Baiyangdian Lake on the aquatic life as of the moment.
